# A New Counter-irritant

**Published:** 1898-09

**Authors:** 


A New Counter-irritant. A patent has recently been applied for to cover the manufacture of a new counter-irritant, which is prepared by percolating powdered capsicum with hot petrolatum.

